# Obesity and thyroid cancer: mechanisms and clinical implications

**DOI:** 10.3389/fnut.2026.1887761

**Published:** 2026-09-29

**Authors:** Haiming Li, Xianqiang Yu

**Affiliations:** 1 Department of Pediatric Urology, Women and Children’s Hospital, Qingdao University, Qingdao, China; 2 Department of Thyroid Disease Diagnosis and Treatment, Qingdao Municipal Hospital (Qingdao Hospital of Rehabilitation University), Qingdao, China

**Keywords:** adipokines, body mass index (BMI), insulin-like growth factor-1 (IGF-1), obesity, thyroid cancer

## Abstract

The global rise in obesity has paralleled the increasing incidence of thyroid cancer, suggesting a potential pathophysiological link. This review comprehensively examines the epidemiological evidence linking obesity to thyroid cancer risk and progression, and elucidates the underlying biological mechanisms. Adipose tissue, particularly visceral fat, functions as an active endocrine organ secreting adipokines, cytokines, and hormones that create a favorable microenvironment for tumor initiation and progression. Key mechanisms include insulin resistance and hyperinsulinemia activating the MAPK and PI3K/AKT pathways, chronic inflammation driven by adipokines, and estrogen-mediated effects. Understanding these pathways provides insights into potential preventive strategies and targeted therapeutic interventions for obese thyroid cancer patients.

## 1 Introduction

The global incidence of thyroid cancer has risen precipitously over recent decades, establishing it as the most common malignancy of the endocrine system (1–10). While improved diagnostic sensitivity, particularly through widespread ultrasound use, has contributed to this trend, it fails to fully explain the epidemiological pattern (11–14). Notably, this surge has occurred in parallel with the escalating global epidemic of obesity, suggesting a potential pathophysiological link. Compelling evidence from numerous large-scale prospective cohort studies and meta-analyses has solidified this connection, demonstrating that obesity is an independent risk factor for thyroid cancer development (15–20). The association exhibits a clear dose-response relationship, with each 5 kg/m^2^ increase in body mass index (BMI) correlating with a significant elevation in risk (21–23). Furthermore, clinical observations indicate that patients with obesity often present with more advanced disease, characterized by larger tumor size and a higher prevalence of lymph node metastasis, hinting that obesity may not only initiate but also promote a more aggressive tumor phenotype (24–29). This convergence of two major public health challenges underscores the critical need to elucidate the biological pathways underpinning their relationship.

The mechanistic link between obesity and thyroid carcinogenesis is multifaceted, rooted in the dysfunctional adipose tissue characteristic of the obese state (Figure 1). Far from being an inert energy reservoir, expanded visceral fat acts as a dynamic endocrine organ, secreting a plethora of bioactive adipokines, cytokines, and hormones that create a systemic environment conducive to tumorigenesis (30–34). The principal mechanisms form an interconnected network: insulin resistance and consequent hyperinsulinemia activate potent mitogenic signaling pathways like PI3K/AKT and MAPK in thyrocytes; a state of chronic low-grade inflammation, driven by pro-inflammatory cytokines such as TNF-α and IL-6, fosters cellular proliferation and genomic instability; and dysregulated adipokine secretion further disrupts normal growth control (32, 35–37). This pro-tumorigenic milieu is compounded by increased aromatase activity in adipose tissue, leading to higher estrogen levels, which may also stimulate thyroid cell growth. These systemic factors likely interact with established driver mutations in thyroid cancer, such as BRAF V600E and RAS, potentially modulating tumor initiation and progression.

**FIGURE 1:**
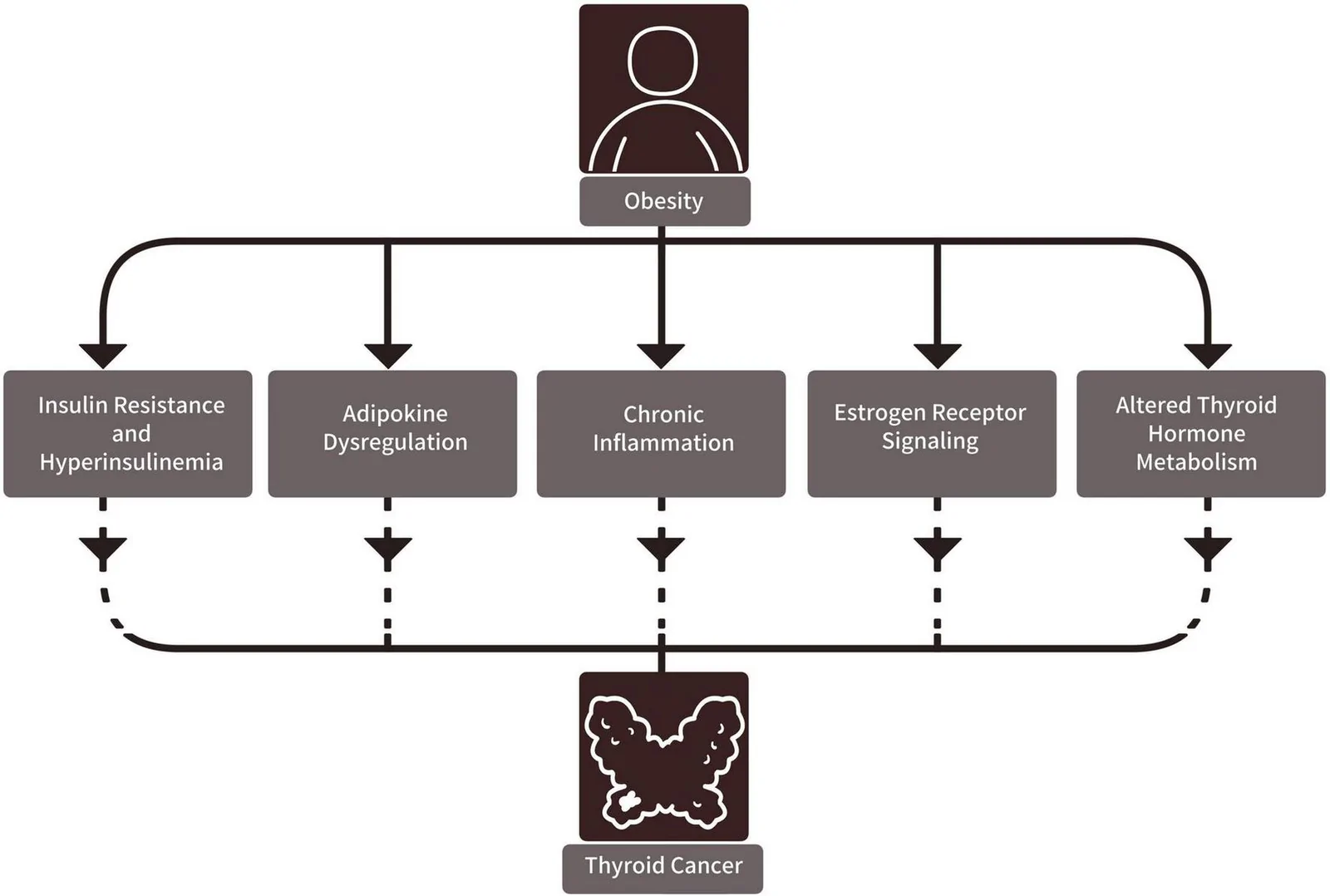
The association between obesity and thyroid cancer.

This review aims to provide a comprehensive synthesis of the current understanding of the impact of obesity on thyroid cancer, with a specific focus on bridging molecular mechanisms to clinical significance. We will begin by consolidating the robust epidemiological evidence establishing obesity as a key risk modifier. The core of the discussion will be a detailed elaboration of the pathophysiological mechanisms, exploring how metabolic dysregulation, inflammation, and hormonal alterations converge to promote thyroid carcinogenesis (Figure 2). Finally, we will translate these mechanistic insights into their clinical implications, examining the influence of obesity on disease presentation, prognostic stratification, therapeutic responses, and long-term patient outcomes. By integrating epidemiological, basic science, and clinical perspectives, this review seeks to highlight the importance of considering metabolic health in the management of thyroid cancer and to identify potential avenues for future research and targeted interventions.

**FIGURE 2:**
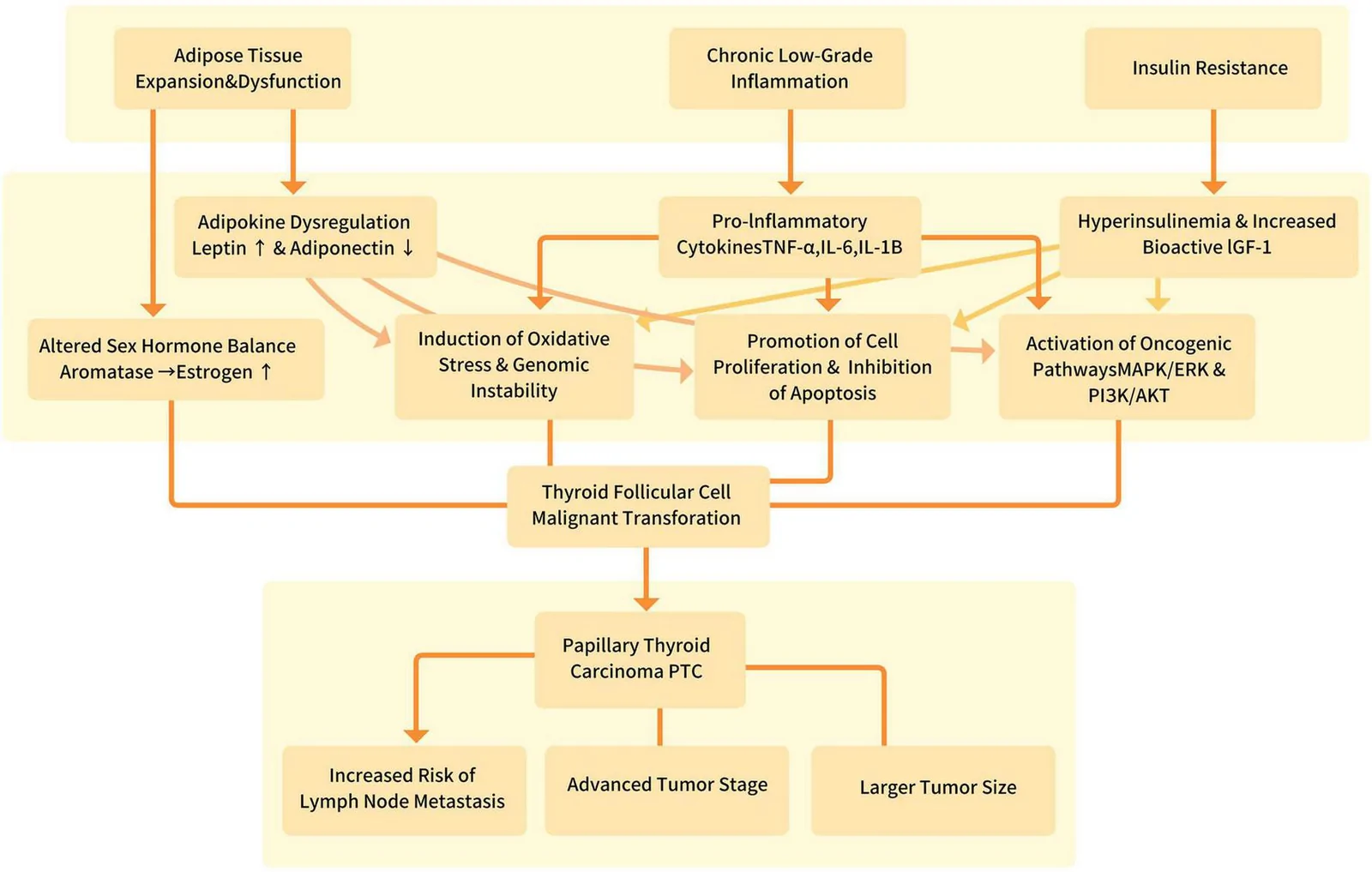
The pathophysiological mechanism of obesity and thyroid cancer.

## 2 Epidemiological evidence

The association between obesity and thyroid cancer is supported by a robust and consistent body of epidemiological evidence derived from large-scale prospective cohorts and comprehensive meta-analyses. A landmark study which pooled individual level data demonstrated a linear dose-response relationship, with each 5 kg/m^2^ increase in body mass index (BMI) associated with a statistically significant 15%–30% elevated risk of developing thyroid cancer (22). This association holds across diverse geographic and ethnic populations, suggesting a fundamental biological link rather than a confounding effect of regional diagnostic practices. Importantly, the risk appears to be stronger in men than in women, a finding that may be partially explained by the differential hormonal milieu. Furthermore, a critical observation from these studies is that the increased risk persists even after adjusting for potential confounders such as smoking status and physical activity, and when analyses are restricted to cancers detected several years after the initial anthropometric assessment (20, 38, 39). The latter point is crucial because it helps to mitigate reverse causal bias, thereby strengthening the inference that obesity is a true causal risk factor for thyroid cancer.

Beyond merely increasing incidence, epidemiological data compellingly link obesity to more advanced and aggressive disease at diagnosis. Multiple hospital-based and population-based studies have consistently reported that obese thyroid cancer patients present with larger tumor diameters, a higher likelihood of extrathyroidal extension, and increased rates of lymph node metastasis compared to their normal-weight counterparts (26–29). This association with adverse pathological features suggests that the procarcinogenic environment of obesity may not only initiate tumor development but also actively fuel tumor progression and invasiveness. The impact of obesity on mortality remains an area of active investigation, with some studies indicating a potential association with higher disease-specific mortality, particularly in advanced cancers. However, the relationship with overall survival is complex and may be influenced by competing health risks associated with obesity. Collectively, this epidemiological foundation provides an indisputable rationale for delving into the pathophysiological mechanisms that underlie this significant public health concern, moving from establishing the “that” to explaining the “how.”

## 3 Pathophysiological mechanisms

### 3.1 Insulin resistance and hyperinsulinemia

The development of insulin resistance represents a fundamental pathophysiological bridge between obesity and thyroid cancer, creating a systemic environment conducive to neoplastic transformation and progression. In the obese state, particularly with visceral adiposity, the expansion of dysfunctional adipose tissue leads to chronic elevations in free fatty acids and pro-inflammatory cytokines such as TNF-α and IL-6 (32, 40–42). These factors impair insulin signaling through multiple mechanisms, including phosphorylation defects in insulin receptor substrate proteins and activation of inflammatory pathways that disrupt normal glucose homeostasis (33, 43–47). The resulting insulin resistance triggers compensatory hyperinsulinemia as pancreatic β-cells increase insulin secretion to maintain euglycemia. This persistent hyperinsulinemia creates a powerful mitogenic environment through both direct receptor-mediated signaling and indirect hormonal modulation that collectively promote thyroid carcinogenesis.

The oncogenic effects of hyperinsulinemia operate through several interconnected mechanisms. Most significantly, elevated insulin levels directly activate key proliferative pathways in thyroid follicular cells through binding to insulin receptors and, at high concentrations, to insulin-like growth factor-1 (IGF-1) receptors (33, 46, 47). This engagement triggers downstream signaling cascades including both the MAPK pathway, which drives cellular proliferation, and the PI3K/AKT pathway, which promotes cell survival and growth. Concurrently, hyperinsulinemia indirectly stimulates thyroid cell proliferation by increasing thyroid-stimulating hormone (TSH) levels and enhancing thyroid gland sensitivity to TSH, a well-established growth factor for thyrocytes (32, 36, 37). Additionally, insulin suppresses hepatic production of IGF-binding proteins, thereby increasing bioavailable IGF-1 that further activates mitogenic signaling. These pathways demonstrate extensive cross-talk with common thyroid cancer driver mutations, such as BRAF V600E, potentially accelerating tumor initiation and progression through synergistic effects on cell cycle regulation and apoptotic resistance.

### 3.2 Adipokine dysregulation

The dysfunctional adipose tissue characteristic of obesity fundamentally alters the systemic endocrine milieu through the dysregulated secretion of adipokines, creating a potent environment for thyroid cancer initiation and progression. In lean individuals, adipose tissue maintains metabolic homeostasis through balanced secretion of adipokines, but obesity induces a state of “adiposopathy” characterized by quantitative and qualitative changes in these signaling molecules (48–51). This imbalance is marked by two principal alterations: the hypersecretion of pro-inflammatory and mitogenic adipokines such as leptin, and the diminished production of protective adipokines like adiponectin. The resulting adipokine disequilibrium establishes a chronic inflammatory and growth-promoting microenvironment that exerts both direct and indirect effects on thyroid follicular cells, effectively lowering the threshold for malignant transformation and supporting tumor development through multiple interconnected pathways.

The mechanisms through which adipokine imbalance promotes thyroid carcinogenesis are multifaceted and synergistic. Leptin, elevated in proportion to adipose mass, acts as a direct mitogen by binding to its specific receptor (Ob-R) expressed on thyroid cells, activating downstream oncogenic signaling cascades including JAK/STAT, MAPK, and PI3K/AKT pathways that collectively stimulate proliferation, inhibit apoptosis, and promote angiogenesis (36, 52–57). Concurrently, the obesity-related reduction in adiponectin, an adipokine with demonstrated anti-proliferative and pro-apoptotic properties, removes a critical inhibitory influence on tumor growth, as adiponectin normally activates AMPK-mediated pathways that counterbalance PI3K/AKT signaling. This dual dysregulation creates a powerful mitogenic stimulus, further amplified by leptin’s ability to stimulate the production of pro-inflammatory cytokines and vascular endothelial growth factor within the tumor microenvironment. Additionally, emerging evidence suggests that adipokines may modulate the expression and activity of key thyroid oncogenes and tumor suppressor genes, potentially influencing tumor aggressiveness and response to therapy, thereby positioning adipokine imbalance as a central mechanism linking obesity to thyroid cancer pathogenesis (58).

### 3.3 Chronic inflammation

The state of chronic low-grade inflammation associated with obesity represents a critical microenvironmental factor in thyroid cancer development and progression. In obesity, hypertrophied adipocytes and infiltrating immune cells within adipose tissue become sources of sustained pro-inflammatory signaling, characterized by elevated systemic levels of cytokines including tumor necrosis factor-α (TNF-α), interleukin-6 (IL-6), and monocyte chemoattractant protein-1 (MCP-1) (59–63). This inflammatory milieu establishes a fertile ground for carcinogenesis through multiple interconnected pathways. Nuclear factor-kappa B (NF-κB) signaling emerges as a central mediator, activated by cytokine receptors and oxidative stress, which subsequently transcriptionally upregulates genes involved in cell proliferation, angiogenesis, and metastasis. Concurrently, immune cell populations within the thyroid microenvironment undergo functional alterations, with increased M2 macrophage polarization and regulatory T cell infiltration creating an immunosuppressive niche that facilitates immune evasion by developing tumor cells.

At the molecular level, chronic inflammation promotes thyroid carcinogenesis through both genomic and non-genomic mechanisms (64, 65). Reactive oxygen and nitrogen species generated by activated inflammatory cells induce DNA damage and genomic instability, increasing the likelihood of oncogenic mutations in genes such as BRAF and RAS. In parallel, cytokine-mediated activation of STAT3 signaling enhances cell survival and proliferation while promoting epithelial-mesenchymal transition, a key process in cancer invasion and metastasis (66). The inflammatory microenvironment further supports tumor progression through the induction of cyclooxygenase-2 (COX-2) and subsequent prostaglandin production, which stimulates angiogenesis and cell growth. This pro-tumorigenic cascade demonstrates significant cross-talk with other obesity-related pathways, particularly insulin resistance and adipokine signaling, creating a self-reinforcing network that collectively drives thyroid cancer initiation, progression, and metastatic potential, thereby establishing chronic inflammation as a fundamental mechanistic link between obesity and thyroid cancer pathogenesis.

### 3.4 Estrogen receptor signaling

The hormonal alterations associated with obesity, particularly in the estrogen signaling pathway, constitute a significant mechanism influencing thyroid cancer development and progression, with distinct implications for gender-specific incidence patterns (67, 68). Adipose tissue serves as a major extra-gonadal site of estrogen production through the activity of aromatase, which converts androgens to estrogens, leading to elevated systemic estrogen levels in obesity. This endocrine environment activates estrogen receptor (ER) signaling in thyroid follicular cells, which express both nuclear estrogen receptors (ERα and ERβ) and membrane-associated G protein-coupled estrogen receptor (GPER) (69–72). The activation of these receptors initiates complex transcriptional and non-genomic signaling events that modulate thyroid cell behavior. Notably, ERα and GPER generally promote proliferative and oncogenic responses, while ERβ often exerts anti-proliferative effects, creating a balance that becomes disrupted in the obese state, favoring pro-tumorigenic pathways.

The oncogenic effects of estrogen receptor signaling in thyroid cancer are mediated through multiple interconnected mechanisms. Ligand-bound ERα translocates to the nucleus and functions as a transcription factor, regulating genes involved in cell cycle progression, such as cyclin D1, and inhibiting pro-apoptotic signals (73–76). Simultaneously, membrane-initiated signaling through GPER rapidly activates downstream kinase cascades including MAPK/ERK and PI3K/AKT, synergizing with other obesity-related pathways such as insulin resistance. This estrogen-mediated signaling demonstrates significant cross-talk with established thyroid cancer drivers. For instance, ER activation can modulate the expression and activity of BRAF and RAS proteins, potentially influencing tumor aggressiveness and therapeutic response (77–80). Furthermore, estrogen signaling contributes to the tumor microenvironment by promoting angiogenesis through vascular endothelial growth factor (VEGF) induction and facilitating inflammatory responses that support cancer progression. The net effect of these estrogen-mediated mechanisms is the creation of a mitogenic environment that lowers the threshold for malignant transformation and supports thyroid tumor growth, providing a physiological basis for the observed gender disparity in thyroid cancer incidence and suggesting potential therapeutic targets for obesity-associated disease.

### 3.5 Altered thyroid hormone metabolism

Obesity induces significant alterations in thyroid hormone metabolism that create a permissive environment for thyroid carcinogenesis, independent of the classic hypothalamic-pituitary-thyroid (HPT) axis regulation (81–84). The low-grade inflammatory state characteristic of obesity, driven by cytokines such as TNF-α and IL-6, directly impacts the expression and activity of deiodinases, the critical enzymes responsible for thyroid hormone activation and inactivation (85–87). Specifically, inflammation suppresses type 1 deiodinase (DIO1) activity in the liver and kidneys while simultaneously enhancing type 3 deiodinase (DIO3) expression in peripheral tissues, creating a state of “low T3 syndrome” or euthyroid sick syndrome (86, 88, 89). This metabolic shift results in reduced bioavailability of biologically active triiodothyronine (T3) at the tissue level, despite normal circulating thyroid hormone levels. The consequent alterations in thyroid hormone signaling disrupt normal cellular metabolism and growth regulation in thyrocytes, establishing a microenvironment conducive to malignant transformation through both direct genomic actions and indirect metabolic effects.

The impact of altered thyroid hormone metabolism on thyroid cancer progression operates through complex molecular mechanisms involving both nuclear receptor-mediated pathways and integrin signaling. Reduced intracellular T3 levels diminish ligand-dependent activation of thyroid hormone receptors (TRs), particularly TRβ1, which normally functions as a tumor suppressor by regulating genes involved in cell cycle arrest and differentiation. This loss of TRβ1-mediated transcriptional control permits uncontrolled proliferation and dedifferentiation of thyroid cells (90–95). Concurrently, the relative increase in thyroxine (T4) levels promotes non-genomic signaling through integrin αvβ3, a plasma membrane receptor that is frequently overexpressed in cancer cells. T4 binding to integrin αvβ3 activates the MAPK and PI3K/AKT pathways, stimulating angiogenesis and tumor cell proliferation. This dual dysregulation, diminished nuclear TR-mediated tumor suppression and enhanced integrin-mediated oncogenic signaling, creates a powerful driver of thyroid cancer progression in the obese state, further exacerbated by the cross-talk between thyroid hormone metabolites and other obesity-related pathways such as insulin resistance and adipokine signaling.

## 4 Clinical implications

The established mechanistic links between obesity and thyroid cancer carry profound implications for clinical practice, fundamentally influencing approaches to risk assessment, preoperative planning, and therapeutic decision-making. From a diagnostic and prognostic perspective, the recognition of obesity as an independent risk factor necessitates its integration into patient stratification models. Clinicians should maintain a higher index of suspicion for thyroid malignancy in patients with obesity, potentially advocating for more vigilant ultrasound surveillance in this population. Furthermore, the association between obesity and aggressive tumor features, such as larger size, extrathyroidal extension, and advanced stage at diagnosis, underscores its value as a prognostic marker. This knowledge should inform preoperative counseling, setting appropriate patient expectations regarding potential surgical complexity and long-term outcomes. The pro-tumorigenic microenvironment created by obesity may also influence the effectiveness of standard therapies, including radioactive iodine uptake, suggesting that metabolic health represents a potentially modifiable factor in treatment planning and response prediction.

Current evidence from large-scale meta-analyses and multicenter cohort studies has substantially strengthened the association between obesity and adverse thyroid cancer outcomes, though the relationship is more nuanced for recurrence and long-term prognosis. A systematic review and meta-analysis demonstrated a statistically significant 25% greater risk of thyroid cancer in overweight individuals and a 55% greater risk in obese individuals compared with normal-weight peers, with each 5-unit increase in BMI associated with a 30% greater risk of thyroid cancer (96). Notably, obesity was significantly positively associated with papillary, follicular, and anaplastic thyroid cancers, while paradoxically showing an inverse association with medullary thyroid cancer (96). A single-center retrospective study of 140 patients also demonstrated that BMI was significantly higher in the BRAFV600E mutation-positive group (*p* < 0.001), and BMI was independently associated with BRAFV600E mutation in both univariate (*p* = 0.007) and multivariate (*p* = 0.026) analyses, suggesting a molecular basis for the observed aggressive phenotype in obese patients (96). However, a Chinese study of 4,369 patients found that while BMI ≥ 24.0 was associated with tumor multifocality and bilaterality, no significant associations were observed with advanced tumor stage, extrathyroidal extension, distant metastasis, or BRAFV600E mutation, indicating that the association may vary across different populations and obesity definitions (97).

The prognostic role of obesity for recurrence and long-term outcomes remains more controversial, highlighting the need for nuanced interpretation of outcome data. A Korean retrospective study of 1,189 papillary thyroid carcinoma patients followed for a median of 8.4 years found no significant associations between BMI quartiles and primary tumor size, extrathyroidal invasion, cervical lymph node metastasis, or distant metastasis (98). Although multifocality and advanced TNM stage showed univariate associations with increasing BMI, these were not significant in multivariate analyses adjusted for age and gender, and no differences in recurrence-free survival were observed across BMI quartiles (log-rank *p* = 0.26) (98). These discrepancies suggest that BMI alone may be an insufficient marker of adiposity-related risk, and other measures may be more relevant for predicting aggressive disease behavior (99). Collectively, the data indicate that while obesity clearly increases thyroid cancer risk and may predispose patients to more aggressive tumor features at presentation, its role as an independent predictor of recurrence and long-term survival remains uncertain, underscoring the need for prospective studies with extended follow-up and standardized obesity metrics to better define clinical implications for risk stratification and surgical management (100).

## 5 Future directions

While significant progress has been made in elucidating the obesity-thyroid cancer axis, several critical avenues for future research remain unexplored and warrant urgent attention. A primary challenge lies in deconvoluting the complex interplay between the various mechanistic pathways (insulin resistance, adipokine imbalance, inflammation, and hormonal signaling) to identify which drivers are predominant and whether they exhibit synergistic effects. Future studies should employ sophisticated multi-omics approaches, integrating genomic, transcriptomic, and proteomic data from well-characterized patient cohorts to define distinct “obesity-related” molecular subtypes of thyroid cancer. Furthermore, a critical, unanswered question is whether the procarcinogenic effects of obesity are reversible. There is a pressing need for prospective, interventional trials to determine if structured weight loss programs, bariatric surgery, or pharmacological management of metabolic syndrome can alter the natural history of thyroid cancer, including reducing incidence, improving treatment response, and decreasing recurrence rates. Establishing a causal, modifiable link would fundamentally shift clinical paradigms.

Translating these mechanistic insights from the bench to the bedside represents the ultimate goal and a fertile ground for innovation. Future efforts must focus on developing and validating integrated prognostic models that incorporate metabolic parameters with traditional TNM staging to improve risk stratification for patients with obesity. From a therapeutic standpoint, research should explore the repurposing of metabolic drugs, such as GLP-1 receptor agonists or SGLT2 inhibitors, as adjuncts to standard cancer care, evaluating their potential to enhance the efficacy of targeted therapies like tyrosine kinase inhibitors. Additionally, investigating the impact of obesity and its associated pathways on the tumor immune microenvironment could reveal novel immunotherapeutic targets or help explain differential responses to immune checkpoint inhibitors. Ultimately, fostering deeper collaboration between endocrinologists, oncologists, and basic scientists will be essential to drive a new era of metabolically informed, personalized management for thyroid cancer patients.

## 6 Conclusion

In conclusion, the compelling epidemiological association between obesity and thyroid cancer is underpinned by a robust and interconnected pathophysiological framework, wherein dysfunctional adipose tissue drives carcinogenesis through a triad of key mechanisms: insulin resistance and hyperinsulinemia activating direct mitogenic signaling, adipokine imbalance fostering a chronic inflammatory and proliferative state, and altered sex hormone metabolism promoting tumor growth. These pathways collectively create a permissive microenvironment that not only increases the risk of malignant transformation but also fuels more aggressive disease biology, thereby explaining the clinical observations of advanced tumor stage and potentially worse outcomes in patients with obesity. This synthesis of mechanisms elevates obesity from a mere comorbid condition to a significant modifiable risk factor and a key determinant of clinical heterogeneity in thyroid cancer, compelling a paradigm shift in patient management that integrates metabolic health optimization through weight management and targeted pharmacological interventions to improve prevention, treatment efficacy, and long-term survival.
